# Alveolar Osteitis: A Comprehensive Review of Concepts and Controversies

**DOI:** 10.1155/2010/249073

**Published:** 2010-06-24

**Authors:** Antonia Kolokythas, Eliza Olech, Michael Miloro

**Affiliations:** Department of Oral and Maxillofacial Surgery, College of Dentistry, University of Illinois at Chicago, 801 South Paulina Street, MC 835, Chicago, IL 60016, USA

## Abstract

Alveolar osteitis, “dry socket”, remains amongst the most commonly encountered complications following extraction of teeth by general dentists and specialists. A great body of literature is devoted to alveolar osteitis addressing the etiology and pathophysiology of this condition. In addition numerous studies are available discussing methods and techniques to prevent this condition. To this date though great controversy still exists regarding the appropriate terminology used for this condition as well as the actual etiology, pathophysiology, and best methods of prevention and treatment. This article is a comprehensive critical review of the available literature addressing the concepts and controversies surrounding alveolar osteitis. We aim to assist the dental health care professional with patient preparation and management of this commonly encountered postoperative condition should be encountered.

## 1. Introduction

Alveolar Osteitis (AO) is a well-known complication after extraction or surgical removal of tooth. Commonly known as “dry socket” this condition remains a common postoperative problem that results in severe pain and repeated practice/hospital visits. The increase in recovery period translates into increased cost to the surgeon as 45% of patients who develop AO typically require multiple postoperative visits in order to manage this condition [1, 2]. However, the exact pathogenesis of AO is not well understood. Many researchers have studied alveolar osteitis, but most concepts are still subject to significant controversy.

## 2. Terminology

Authors do not agree on terminology for this complication. “Dry socket” was first described in the literature in 1896 by Crawford [3]. Since then, other terms have been used to refer to this complications, such as “alveolar osteitis”, “alveolitis”, “localized osteitis”, “alveolitis sicca dolorosa”, “localized alveolar osteitis”, “fibrinolytic alveolitis”, “septic socket”, “necrotic socket”, and “alveolalgia”, among others [4–6]. Birn, whose series of articles provided a better understanding of the pathophysiology [7–9], labeled the condition fibrinolytic alveolitis. Although most authors have accepted Birn's theories, the term fibrinolytic osteitis is the least used in the literature [4–6]. “Dry socket”, which is the generic term, and “alveolar osteitis” are more commonly used terms.

## 3. Definition

Eighteen definitions of AO have been reported [4]. The most recent defines AO as “postoperative pain inside and around the extraction site, which increases in severity at any time between the first and third day after the extraction, accompanied by a partial or total disintegrated blood clot within the alveolar socket with or without halitosis” [4]. The literature is abundant with diverse descriptive definitions for AO, which probably leads to the discrepancy in the diagnostic criteria. Several authors have agreed that pain and empty alveolus are found in all patients with AO [7, 10–13]. Other defining factors that have been reported are radiating pain towards the ear and temporal region [14, 15], rare maxillary involvement in ocular and frontal regions [7], halitosis [14, 16], seldom low-grade fever [14, 15], inflamed gingival margin [17], bare bone [17], ipsilateral regional lymphadenopathy [14, 15], and grayish discharge [18].

## 4. Incidence

The frequency of AO has been the subject of many articles in the literature. The lack of objective clinical criteria leads to considerable variability in the reported frequency of AO. Poor study design, miscalculation of data, insufficient sample, or introduction of variables could also contribute to the variability that has been reported in the literature. For routine dental extractions, the incidence of AO has been reported in the range 0.5% to 5% [11, 12, 48, 49]. The incidence of AO after extraction of mandibular third molars varies from 1% to 37.5% [28, 50]. It has been well documented that surgical extractions result in about 10 times higher incidence of AO [4].

## 5. Onset

Throughout the literature the onset of AO is considered to occur 1–3 day after tooth extraction [14, 21, 26]. 95–100% of all cases of AO have been reported within a week [11].

## 6. Etiology

The exact pathogenesis of AO is not well understood. Birn's classic series of articles between 1963 and 1977 provided a better understanding of the likely pathophysiology [7–9]. Birn suggested that the etiology of AO is an increased local fibrinolysis leading to disintegration of the clot. The fibrinolysis is the result of plasminogen pathway activation, which can be accomplished via direct (physiologic) or indirect (nonphysiologic) activator substances [7]. Direct activators are released after trauma to the alveolar bone cells. Indirect activators are elaborated by bacteria. The fibrinolytic activity is local because initial absorption of plasminogen into the clot limits the activity of plasmin. In fact, it was found that active plasmin is inactivated in the general circulation by antiplasmins [51]. Birn and others have further reviewed the local differences in the fibrinolytic activity between body tissue and found higher fibrinolytic activity with bone and uterine tissues, in comparison to skeletal muscle, kidney, heart, brain, liver, spleen, lung, and thyroid tissues [52, 53]. But the factors capable of triggering fibrinolysis are more ambiguous. There have been numerous studies published over the years discussing the contributing or risk factors for development of AO and a review of the most commonly involved ones is provided below and all are presented cumulatively in Table 1.

## 7. Contributing/Risk Factors

### 7.1. Surgical Trauma and Difficulty of Surgery

Most authors agree that surgical trauma and difficulty of surgery play a significant role in the development of AO [6, 7, 27, 34]. This could be due to more liberation of direct tissue activators secondary to bone marrow inflammation following the more difficult, hence, more traumatic extractions [35]. Surgical extractions, in comparison to nonsurgical extractions, result in a 10-fold increase incidence of AO [5]. Lilly et al. [10] found that surgical extractions involving reflection of a flap and removal of bone are more likely to cause AO.

### 7.2. Lack of Operator Experience

Many studies claim that operator's experience is a risk factor for the development of AO. Larsen [2] concluded that surgeon's inexperience could be related to a bigger trauma during the extraction, especially surgical extraction of mandibular third molars. Alexander [6] and Oginni et al. [36] both reported a higher incidence of AO following extractions performed by the less experienced operators. Therefore the skill and experience of the operator should be taken into consideration.

### 7.3. Mandibular Third Molars

It has been shown that alveolar osteitis is more common following the extraction of mandibular third molars [54, 55]. Some authors believe that increased bone density, decreased vascularity, and a reduced capacity of producing granulation tissue are responsible for the site specificity [54]. However, there is no evidence suggesting a link between AO and insufficient blood supply. The area specificity is probably due to the large percentage of surgically extracted mandibular molars and may reflect the effect of surgical trauma rather than the anatomical site [35].

### 7.4. Systemic Disease

Some researchers have suggested that systemic disease could be associated with alveolar osteitis [7, 10]. One article proposed immunocompromised or diabetic patients being prone to development of alveolar osteitis due to altered healing [5]. But no scientific evidence exists to prove a relationship between systemic diseases and AO.

### 7.5. Oral Contraceptives

Oral contraceptive is the only medication associated with developing AO. Oral contraceptives became popular in 1960s and studies conducted after 1970s (as opposed to studies prior to 1960s) show a significant higher incidence of AO in females [38, 40, 41]. Sweet and Butler [20] found that this increase in the use of oral contraceptives positively correlates with the incidence of AO. Estrogen has been proposed to play a significant role in the fibrinolytic process. It is believed to indirectly activate the fibrinolytic system (increasing factors II, VII, VIII, X, and plasminogen) and therefore increase lysis of the blood clot [37]. Catellani et al. [56] further concluded that the probability of developing AO increases with increased estrogen dose in the oral contraceptives. One author [41] even suggested that in order to reduce the risk of AO, hormonal cycles should be considered when scheduling elective exodontia.

### 7.6. Patient's Gender

Many authors claim that female gender, regardless of oral contraceptive use, is a predisposition for development of AO. MacGregor [12] reported a 50% greater incidence of AO in women than that in men in a series of 4000 extractions, while Colby [34] reported no difference in the incidence of AO associated with gender.

### 7.7. Smoking

Multiple studies demonstrated a link between smoking and AO. A dose dependent relationship between smoking and the occurrence of alveolar osteitis has been reported. Among a total of 4000 surgically removed mandibular third molars, patients who smoked a half-pack of cigarettes a day had a four- to five-fold increase in AO (12% versus 2.6%) when compared to nonsmokers. The incidence of AO increased to more than 20% among patients who smoked a pack per day and 40% among patients who smoked on the day of surgery [57]. Whether a systemic mechanism or a direct local affect (heat or suction) at the extraction site is responsible for this increase is unclear [35]. Blum speculated that this phenomenon could be due to the introduction of foreign substance that could act as a contaminant in the surgical site [4].

### 7.8. Physical Dislodgement of the Clot

Although a very commonly discussed theory, no evidence exists in the literature verifying that physical dislodgement of the blood clot caused by manipulation or negative pressure created via sucking on a straw would be a major contributor to AO [4].

### 7.9. Bacterial Infection

Most studies support the claim that bacterial infections are a major risk for the development of AO. It has been shown that the frequency of AO increases in patients with poor OH [46], preexisting local infection such as periocoronitis and advanced periodontal disease [45]. Attempts have been made to isolate specific causative organisms. A possible association of Actinomyces viscosus and *Streptococcus mutans* in AO was studied by Rozantis et al. [58], where they demonstrated delayed healing of extraction sites after inoculation of these microorganisms in animal models. Nitzan et al. [59] observed high plasmin-like fibrinolytic activities from cultures of Treponema denticola, a microorganism present in periodontal disease. Catenalli [24] studied bacterial pyrogens *in vivo* and postulated that they are indirect activators of fibrinolysis.

### 7.10. Excessive Irrigation or Curettage of Alveolus

It has been postulated that excessive repeated irrigation of alveolus might interfere with clot formation and that violent curettage might injure the alveolar bone [7]. However, the literature lacks evidence to confirm these allegations in the development of AO.

### 7.11. Age of the Patient

Little agreement can be found as to whether age is associated with peak incidence of AO. The literature supports the general axiom that the older the patient, the greater the risk [6]. Blondeau et al. [60] concluded that surgical removal of impacted mandibular third molars should be carried out well before age of 24 years, especially for female patients since older patients are at greater risk of postoperative complications in general.

### 7.12. Single Extraction versus Multiple Extractions

Limited evidence exists indicating higher prevalence of AO after single extractions versus multiple extractions [11, 12]. In one study, AO prevalence was 7.3% following single extractions and 3.4% following multiple extractions [35]. This difference could possibly be due to less pain tolerance in patients with single extractions compared to patients with multiple extractions whose teeth have deteriorated to such an extent that multiple extractions are needed [11]. Moreover, multiple extractions involving periodontally diseased teeth may be less traumatic.

### 7.13. Local Anesthetic with Vasoconstrictor

It has been suggested that the use of local anesthesia with vasoconstrictors increases the incidence of AO. Lehner [43] found that AO frequency increases with infiltration anesthesia because the temporary ischemia leads to poor blood supply. However, the studies that followed indicated that ischemia lasts for one to two hours and is followed by reactive hyperemia, which makes it irrelevant in the disintegration of the blood clot [7, 44]. One study reported no significant difference in AO prevalence following extraction of teeth requiring infiltration anesthesia versus regional block anesthesia with vasoconstrictor [35]. It is currently accepted that local ischemia due to vasoconstrictor in local anesthesia has no role in the development of AO.

### 7.14. Saliva

A few authors have argued that saliva is a risk factor in the development of AO [23, 25]. However, no firm scientific evidence exists to support this claim. Birn found no evidence that saliva plays a role in AO [7].

### 7.15. Bone/Root Fragments Remaining in the Wound

Some authors have suggested that bone/root fragments and debris remnants could lead to disturbed healing and contribute to development of AO [4, 7]. Simpson, in his study, showed that small bone/root fragments are commonly present after extractions and these fragments do not necessarily cause complications as they are often externalized by the oral epithelium [47].

### 7.16. Flap Design/Use of Sutures

Some previous literature claims that design of a flap and the use of sutures affect the development of AO [38]. However, more recent studies found little evidence to prove such relationship [84]. In the absence of any significant evidence, it is reasonable to assume that these are not major contributing factors [6].

## 8. Prevention

Since AO is the most common postoperative complication after extraction, many researchers have attempted to find a successful method for prevention. Numerous methods and techniques are proposed throughout the existing literature to assist with prevention of AO. However, this area remains a controversial topic as no single method has gained universal acceptance. The most popular of these techniques are discussed below and are cumulatively presented in Table 2.

### 8.1. Systemic Antibiotics

Systemic antibiotics reported to be effective in the prevention of AO include penicillins [61, 64], clindamycin [61, 62], erythromycin [62], and metronidazole [21, 30]. The routine use of systemic pre- and/or postoperative antibiotics prophylatically is disputed though due to development of resistant bacterial strains, possible hypersensitivity, and unnecessary destruction of host commensals [4, 63].

### 8.2. Topical Antibiotics

A great number of studies have been performed in order to test the effectiveness of topical medicaments in preventing AO. The antibiotics studied have been used alone or in combination with differing doses and formulations. As expected there is a lack of consistency and very few studies are in agreement. Amongst the many antibiotics studied, topical tetracycline has shown promising results [32, 66, 68, 70]. The reported method of delivery included powder, aqueous suspension, gauze drain, and Gelfoam sponges (preferred). However, side-effects including foreign body reactions have been reported with the application of topical tetracycline [67, 69]. In one study, myospherulosis resulted from petroleum-based carrier used in tetracycline-hydrocortisone combination [65]. Zuniga and Leist reported a case in which the patient developed a nerve dysesthesia six months after mandibular third molar extraction due to use of medications in the socket [69]. One author suggested that putting virtually anything into the alveolus, including plain Gelfoam, will result in at least a slight improvement in the incidence of AO [14]. 

### 8.3. Chlorhexidine

Several studies have reported that the pre- and perioperative use of 0.12% chlorhexidine decreases the frequency of AO after mandibular third molar removal [31, 42, 71, 72]. Ragno et al. [85] found as much as 50% reduction in the incidence of AO in patients who prerinsed with chlorhexidine solution. Caso et al. [73] after a meta-analysis of the available studies concluded that 0.12% chlorhexidine rinse on the day of surgery and for several days thereafter is beneficial. 

### 8.4. Para-Hydroxybenzoic Acid

Early literature reported that the topical use of para-hydroxybenzoic acid (PHBA), an antifibrinolytic agent, in extraction wounds decreased the incidence of AO [74, 76]. PHBA is available on the market as a component of Apernyl (Bayer AG, Germany), an alveolar cone that consists of acetylsalicylic acid and PHBA. Apernyl was investigated by some researchers [19, 75], who claimed its success, but also noted that it inhibited bone healing in animal studies. In these studies, it is not possible to attribute the reported findings to antifibrinolytic properties of PHBA or perhaps to the anti-inflammatory properties of aspirin. In addition, PHBA has been reported to have some antimicrobial properties [15]. Aspirin in contact with bone has been found to cause local irritation and subsequent inflammation of the socket [86]. 

### 8.5. Tranexamic Acid

Tranexamic acid (THA), an antifibrinolytic agent, has been speculated to prevent AO when applied topically in the extraction socket [77]. But a study by Gersel-Pedersen [78] did not show a significant reduction in the incidence of AO when compared to a placebo group. Local plasminogen inactivation alone was insufficient to cease the development of AO. 

### 8.6. Polylactic Acid

Polylactic acid (PLA), a clot supporting agent, is a biodegradable ester that once was thought to be the ultimate solution for the prevention of AO. It was suggested that PLA would provide a stable support for the blood clot and subsequent granulation and osteoid tissue. A study by Brekke et al. [27] reported a significant reduction in AO when PLA was used. However, follow-up studies failed to support the success of PLA [33, 67]. Complications were observed and the reported incidence of AO was actually higher when PLA was used. PLA is still available today under the brand name of DriLac (Osmed, Inc, Costa Mesa, CA, USA). 

### 8.7. Steroids

Lele in 1969 found corticosteroid use to decrease postoperative complications but failed to prevent development of AO [80]. More recent studies showed that topical application of an emulsion of hydrocortisone and oxytetracycline significantly reduced AO after impacted mandibular molar removal [14, 79]. “Unfortunately, the contribution of antibiotic cannot be separated from that caused by the steroid” [4].

### 8.8. Eugenol Containing Dressing

Some authors have promoted the use of eugenol containing dressing to prevent development of AO [81]. However, irritant local effect of eugenol and the delay in wound healing due to prophylactic packing has been well documented in the literature and may be difficult to justify its use to prevent AO [6, 39, 76].

### 8.9. Lavage

Some authors have suggested copious intraoperative lavage to reduce the incidence of AO. Butler and Sweet [22] reported significant reduction in AO when 175 mL lavage was used as compared to 25 mL lavage. However, in another study, the same researchers increased the lavage volume to 350 mL [20]. No significant differences were observed relative to the effect of 175 mL versus 350 mL lavage volume on the incidence of AO.

### 8.10. 9-Aminoacrinide

There is one study in which 9-aminoacridine, an antiseptic agent, was evaluated for its effectiveness in reducing the incidence of AO but was found to be ineffective [29].

### 8.11. Sterile Gloves

The use of sterile gloves instead of clean nonsterile gloves has not demonstrated a decrease in the incidence of AO and therefore not necessary [82, 83].

## 9. Management

The management of AO is less controversial than its etiology and prevention. A few authors have referred to the “treatment” of AO [21, 34, 87]. Recommending “treatment” appears to be misleading as the condition cannot be treated as long as the etiology has not been firmly established [4]. Most agree that the primary aim of dry socket management, as indicated by Fazakerley [88], is pain control until commencement of normal healing, and in the majority of cases local measures are satisfactory. In some instances, systemic analgesics or antibiotics may be necessary or indicated. The use of intra-alveolar dressing materials is widely suggested in the literature [13, 15, 89], although it is generally acknowledged that dressings delay healing of the extraction socket [76]. Different medicaments and carrier systems are commercially available with little scientific evidence to guide a selection process as demonstrated above. As the various formulations are reviewed, it becomes apparent that all of them are simply varying combinations of perhaps 18 different ingredients [6]. Alvogyl (Septodent, Inc, Wilmington, DE) has been widely used in the management of AO and is frequently mentioned in the literature. Alvogyl contains butamben (anesthetic), eugenol (analgesic), and iodophorm (antimicrobial). Some authors [90, 91] noted retardation of healing and inflammation when the sockets were packed with Alvogyl. They did not recommend its use in extraction sockets.

## 10. Conclusions

Despite many years of research, little progress has been made in addressing this commonly encountered and unpleasant postoperative condition in patients. The literature regarding alveolar osteitis is not consistent and often conflicting. Studies are poorly designed, have varying designs and statistical biases, lack analysis, or consist of individual opinions. The full etiology of alveolar osteitis has not been established and varying descriptive definitions and diagnostic criteria exist to explain alveolar osteitis. This lack of simplistic answer, according to one author, is because the initiation of fibrinolytic process appears to be related to an interfacing of multiple independent factors [6]. Research attempting to prevent this complication yields no single universally acceptable method or success. However, a multitude of intra-alveolar medicaments are suggested in the literature and are available on the market. Even though complications/severe reactions from preparations placed in the socket are rare, almost all have reported some negative reactions. If adverse reactions do occur, the current body of literature does not provide enough support for the treating practitioner. The formula to management of this complication should begin with patient education and patients with identifiable risk factors should be informed in detail about this anticipated complication. Further investigations and well-designed studies are necessary to draw firm conclusions and to clarify this complication.

## Figures and Tables

**Table 1:** Proposed risk factors and associated literature.

Risk factors	Authors supporting	Authors refuting
Trauma/difficult extraction	Birn (1973) [7]	Meyer (1971)
	Hellem & Nordenram (1973)	Ritzau & Swangsilpa (1977) [19]
	Lilly et al. (1974) [10]	Sweet & Butler (1978) [20]
	Keskitalo & Persson (1975)	Rood & Murgatroyd (1979) [21]
	Butler & Sweet (1977,1985) [22]	Krekmanov & Hallander (1980) [23]
	Catellani (1979) [24]	Schofield & Warren (1981)
	MacGregor (1979)	Krekmanov (1981) [25]
	Matthews (1982)	Nitzan (1983) [26]
	Brekke et al. (1983,1986) [27]	Heasman & Jacobs (1984) [28]
	Johnson and Blanton (1988) [29]	Field et al. (1985) [11]
	Fridrich & Olson (1990) [14]	Barclay (1987) [30]
	Larsen (1991) [31]	Swanson (1989) [32]
	Hooley & Golden (1995) [33]	MacGregor (1990)
	Colby (1997) [34]	
	Alexander (2000) [6]	
	Torres-Lagares et al. (2005) [5]	
	Nusair & Younis (2007) [35]	

Inexperienced surgeon	Sisk et al. (1986)	Nusair & Younis (2007) [35]
	Larsen (1991) [31]	
	Herpy & Goupil (1991)	
	Larsen (1992) [2]	
	Alexander (2000) [6]	
	Oginni et al. (2003) [36]	

Gender (female)	MacGregor (1968) [12]	Catellani (1979) [24]
	Field et al. (1985) [11]	Heasman & Jacobs (1984) [28]
	Herpy & Goupil (1991)	Larsen (1992) [2]
	Alexander (2000) [6]	Colby (1997) [34]
		Nusair & Younis (2007) [35]

Oral contraceptives	Ygge et al. (1969) [37]	Barclay (1987) [30]
	Schow (1974) [38]	Larsen (1992) [2]
	Butler & Sweet (1977) [22]	Chapnick & Diamond (1992) [39]
	Sweet & Butler (1977,1978) [20, 40]	
	Fridrich & Olson (1990) [14]	
	Cohen & Simecek (1995) [41]	
	Hermesch et al.(1998) [42]	
	Alexander (2000) [6]	

Smoking	Sweet & Butler (1978) [20]	Barclay (1987) [30]
	Meechan et al. (1988)	Johnson and Blanton (1988) [29]
	Larsen (1992) [2]	
	Nusair & Younis (2007) [35]	

Increased age	Birn (1973) [7]	Catellani (1979) [24]
	Herpy & Goupil (1991)	Schofield & Warren (1981)
	Alexander (2000) [6]	Heasman & Jacobs (1984) [28]
		Fridrich & Olson (1990) [14]
		Larsen (1992) [2]
		Nusair & Younis (2007) [35]

Flap design/suture	Rud et al. (1963)	Belinfante et al. (1973)
	Schow (1974) [38]	Sweet & Butler (1978) [20]
	Martis et al. (1978)	
Vasoconstrictors in local anesthetics	Lehner (1958) [43]	Meyer (1971)
		Birn (1973) [7]
		Tsirlis et al. (1992) [44]
		Alexander (2000) [6]

Saliva	Krekmanov & Hallander (1980) [23]	Birn (1973) [7]
	Krekmanov (1981) [25]	Nitzan (1983) [26]
		Alexander (2000) [6]

Bacterial involvement	MacGregor (1968) [12]	Martis et al. (1978)
	Rud (1970) [45]	
	Birn (1973) [7]	
	Rood & Murgatroyd (1979) [21]	
	Krekmanov & Hallander (1980) [23]	
	Krekmanov (1981) [25]	
	Nitzan (1983) [26]	
	Swanson (1989) [32]	
	Peñarrocha-Diago et al. (2001) [46]	

Single extraction (versus multiple)	Nusair & Younis (2007) [35]	MacGregor (1968) [12]
		Birn (1973) [7]
		Tsirlis et al. (1992) [44]
		Field et al. (1985) [11]

Bone/root fragments	Birn (1973) [7]	Simpson (1969) [47]
	Blum (2002) [4]	

**Table 2:** Proposed techniques for decreasing risk for development of AO and related literature.

Method/technique	Authors supporting	Authors refuting
Systemic antibiotics	Laird et al. (1972) [61]	Alexander (2000) [6]
	Rood & Murgatroyd (1979) [21]	Blum (2002) [4]
	Bystedt et al. (1980) [62]	Ataoǧlu et al. (2008) [63]
	Krekmanov & Nordenram (1986) [64]	
	Barclay (1987) [30]	

Topical antibiotics	Hall et al. (1971)	Lynch & Newland (1984) [65]
	Davis et al. (1981) [66]	Moore & Brekke (1990) [67]
	Sorensen & Preisch (1987) [68]	Zuniga & Leist (1995) [69]
	Swanson (1989) [32]	
	Akota et al. (1998) [70]	

Antiseptic Mouthrinse/lavage	Butler & Sweet (1977) [22]	Sweet & Macynski (1985)
	Tjernberg (1979) [71]	Berwick & Lessin (1990) [72]
	Berwick & Lessin (1990) [72]	
	Larsen (1991) [31]	
	Hermesch et al.* (1998)* [42]	
	Alexander (2000) [6]	
	Blum (2002) [4]	
	Caso et al. (2005) [73]	

PHBA	Birn (1972) [8, 74]	Kjellman (1973) [75]
	Ritzau & Swangsilpa (1977) [19]	
	Ritzau & Therkildsen (1978)	
	Schatz et al. (1987) [76]	

Tranexamic acid	Ritzau (1973) [77]	Gersel-Pedersen (1979) [78]

Polylactic acid	Brekke et al. (1986) [27]	Moore & Brekke (1990) [67]
		Hooley & Golden (1995) [33]

Steroids	Rutledge & Marcoot (1984) [79]	Lele (1969) [80]
	Fridrich & Olson (1990) [14]	

Eugenol containing dressing	Bloomer (2000) [81]	Schatz (1987) [76]
		Alexander (2000) [6]

9-aminoacridine	n/a	Johnson and Blanton (1988) [29]

Sterile gloves	n/a	Cheung et al. (2001) [82]
		Adeyemo et al. (2005) [83]
